# Myeloid-Derived Suppressor Cells: Not Only in Tumor Immunity

**DOI:** 10.3389/fimmu.2019.01099

**Published:** 2019-05-15

**Authors:** Graham Pawelec, Chris P. Verschoor, Suzanne Ostrand-Rosenberg

**Affiliations:** ^1^Department of Immunology, University of Tübingen, Tübingen, Germany; ^2^Health Sciences North Research Institute, Sudbury, ON, Canada; ^3^Department of Health Research Methods, Evidence and Impact, McMaster University, Hamilton, ON, Canada; ^4^Department of Pathology and Huntsman Cancer Institute, University of Utah, Salt Lake City, UT, United States

**Keywords:** MDSC, cancer immunity, obesity, autoimmunity, aging, transplantation, infectious disease

## Abstract

Since the realization that immature myeloid cells are powerful modulators of the immune response, many studies on “myeloid-derived suppressor cells” (MDSCs) have documented their ability to promote tumor progression in melanoma and other cancers. Whether MDSCs are induced solely pathologically in tumorigenesis, or whether they also represent physiological immune control mechanisms, is not well-understood, but is particularly important in the light of ongoing attempts to block their activities in order to enhance anti-tumor immunity. Here, we briefly review studies which explore (1) how best to identify MDSCs in the context of cancer and how this compares to other conditions in humans; (2) what the suppressive mechanisms of MDSCs are and how to target them pharmacologically; (3) whether levels of MDSCs with various phenotypes are informative for clinical outcome not only in cancer but also other diseases, and (4) whether MDSCs are only found under pathological conditions or whether they also represent a physiological regulatory mechanism for the feedback control of immunity. Studies unequivocally document that MDSCs strongly influence cancer outcomes, but are less informative regarding their relevance to infection, autoimmunity, transplantation and aging, especially in humans. So far, the results of clinical interventions to reverse their negative effects in cancer have been disappointing; thus, developing differential approaches to modulate MSDCs in cancer and other diseases without unduly comprising any normal physiological function requires further exploration.

## Introduction

Myeloid cells encompassing monocytes, macrophages, dendritic cells, polymorphonuclear granulocytic cells and others are continuously generated from hematopoietic stem cells through multi-step differentiation processes. The presence of cancer can skew hematopoiesis toward myelopoiesis, probably mediated by pro-inflammatory factors (1). Interestingly, similar phenomena occur in overtly cancer-free aging, presumably for the same reasons (2). Immature myeloid cells at certain stages of differentiation may act in an immunosuppressive manner and are therefore designated “myeloid-derived suppressor cells” (MDSCs). MDSCs have been most intensively studied in the context of cancer (3). They are a very heterogeneous group of mononuclear and polymorphonuclear myeloid cells, normally present at very low numbers in healthy individuals, but may accumulate under disease conditions (4)—or potentially during natural aging (5, 6) or with psychological stress (7). These influences, in addition to heterogeneity in the differentiation trajectory for myeloid lineage cells, means that there are no validated unique phenotypic markers for MDSCs and they can only be unequivocally identified using functional assays (8). Unfortunately, these biological assays have many drawbacks in terms of reproducibility, standardization, requirements for large amounts of cells, etc. Therefore, despite the lack of robust associations between phenotypes and function, many studies rely on the former as a surrogate for the latter. The field is further complicated by inherent differences between humans and animal models, mostly mice, which make preclinical studies challenging (9, 10). Hence, in this article, we will attempt to briefly review (1) how best to identify MDSCs in humans; (2) what are their suppressive mechanisms and how to target them pharmacologically; (3) if their levels of MDSCs informative for clinical outcomes; and (4) whether MDSCs are only important in pathology or whether they also represent physiological regulatory mechanisms for the feedback control of immunity. In this latter section, relying mostly on studies in mouse models which can be experimentally manipulated, a basis is established for translating animal data to areas less well-documented than cancer in humans. Because the role of MDSCs in cancer has been subject to considerable recent scrutiny [reviewed in (11, 12)], we will only very briefly cover this issue and focus more on the importance of MDSCs in other conditions.

## Identification of MDSCs

### Characteristics of MDSCs

MDSCs were first phenotypically identified in tumor-bearing mice by their expression of CD11b and Gr-1 (13, 14). This is not possible in humans because there is no Gr-1 homolog. As a simplification, in humans, functional MDSCs are generally recognized to be either mononuclear and monocytic (M-MDSCs, expressing surface CD14), or polymorphonuclear and granulocytic (PMN-MDSCs, expressing CD15) (8). Accurate phenotypic characterization requiring functional studies is challenging in humans, not least because healthy people possess very few MDSCs and accessing sufficient amounts of blood from patients, especially those with cancer, is not trivial. For multi-center studies there is the additional limitation that the requirement for cryopreservation of samples means that only M-MDSCs can be readily analyzed because PMN-MDSC do not readily survive freezing (15). Furthermore, as the source of this material is mostly peripheral blood, the method of cell isolation, freezing and storage adds further variation. Perhaps because of this, and the lack of the human CD11b-Gr-1 murine standard, it is commonly the experience that individual laboratories are using very different approaches to phenotyping and functional testing. In the context of applying MDSC data for use as biomarkers for clinical prognosis and prediction, this raises concerns about routine applicability of any MDSC methodology. Many efforts have been addressed toward attempting to resolve this issue. Recommendations based on multi-center collaborative studies are beginning to make some inroads into solving this problem, for example, by paralleling subtypes of MDSCs defined phenotypically with their suppressive activity in functional assays (8). However, the problem is compounded by the fact that sorting MDSCs for use in functional tests is itself problematic and while several different approaches remain in use, a great deal of heterogeneity persists in the literature. Separating cells by magnetic bead sorting is limited by the small number of markers that can be used but multi-parametric fluorescence-activated cell sorting (FACS) is expensive, slow and can alter cell characteristics. It seems safe to conclude that there is no optimal approach to isolate MDSCs and attempts to increase the sophistication of phenotyping and biological assays of suppression will remain challenging. Knowledge of the mechanisms employed by MDSCs to suppress immune responses may allow biochemical and/or genetic analyses to circumvent cumbersome phenotyping and functional assays in the future (see following sections).

Efforts to better characterize the surface phenotype of MDSCs have included the use of CyTOF to increase the number of possible subsets detectable. Unfortunately, CyTOF cannot sort viable cells for functional testing, although this may not be necessary when the desire is only to establish biomarkers relevant to cancer patient survival. Few data were available at the time of writing. A pilot study used CyTOF to examine extended phenotypes in 5 melanoma patients (16), and in our own pilot study of 27 stage IV melanoma patients we were unable to identify an extended phenotype that correlated with overall survival better than the basic M-MDSC phenotype CD14^+^CD11c^+^HLA-DR^−/lo^, despite including over 30 markers (17). However, this pilot study still included only very few patients and work is ongoing. Considering that monocytes could themselves be viewed as immature macrophages, it is not surprising that phenotypes close to the classical monocyte phenotype delineate populations of cells with regulatory activities (18). Multi-parametric FACS has also increased in sophistication since its introduction, as illustrated for example in the report of the CIMT multi-center phenotyping harmonization study (19), but these more extended phenotypes may also not prove any better than the simple monocyte phenotype. Thus far, the type of transcriptomics approach so widely used for analyzing T and B cell populations yielded relatively sparse information on potential gene expression patterns in the different populations of MDSCs (20), so progress might be possible at this level in the future. The increasing availability of databases and analytical algorithms to assess the presence of immune cells within tumors encourage the belief that this approach will soon yield valuable insights (21).

### Induction of MDSCs

What is clear from both animal and human studies is the strong influence of an inflammatory or anti-inflammatory microenvironment on the differentiation of myeloid precursors into functional cells (11, 14, 22). Much of what we know about the induction of M-MDSCs in humans derives from *in vitro* experiments sequentially culturing monocytes with different cytokines and chemokines to mimic the inflammatory/anti-inflammatory microenvironment, and then analyzing the phenotypes and functions of the derived cells (23). Modifications of these approaches include cultures containing tumor cells to induce MDSCs (24, 25), which also provide an opportunity to investigate how to prevent their induction, as for example in Janssen et al. (26). These properties can then be compared with cells from patients or healthy controls.

Such monocyte-derived cells can be made to differentiate into immune stimulatory cells (predominantly dendritic cells, DCs) or tolerogenic cells reminiscent of MDSCs, depending on the culture protocol. Many experiments expose monocytes to a mixture of GM-CSF and IL-4 for a few days to generate activated immature cells which can then be caused to mature into DCs by adding inflammatory cytokines. However, if IL-10 is present from the beginning of culture, resulting cells maintain high levels of CD14 but downregulate HLA-DR, a classic MDSC phenotype, and also express characteristic molecules like glucocorticoid-induced-tumor-necrosis-factor-receptor-related-protein (GITR) (27). Thus, at least some of the anti-inflammatory and immunosuppressive effects of IL-10 may be mediated in this manner. It is the balance of soluble factors and other stimuli during the differentiation of the precursors that clearly affects the final outcome, complex to examine *in vitro*, near impossible *in vivo* in humans. Nonetheless, *in vitro* studies can point to targets addressable in patients, for example altering the PGE_2_:COX2 ratio (28), as further discussed below.

## Measurement and Mechanisms of Suppression

### Assays and Mechanisms of Suppression

By separating candidate M-MDSCs on the basis of their phenotype and testing each subset for suppressive activity, it was hoped to identify the most biologically relevant phenotype (with the caveat that *in vitro* suppressor assays will still only be biomarkers that need to be associated with a robust clinical outcome in order to be meaningful). M-MDSCs produce multiple molecules that could be candidates for mediating suppression. Of these, the “metabolic inhibitors” arginase-1 (ARG-1) and indolamine-2,3-dioxygenase- (IDO), catabolizing arginine and tryptophan, respectively, have been extensively investigated, but many other molecules such as the cytokines IL-10 and TGF-ß, as well as nitric oxide (NO) are likely to be involved as well (10, 29). Whereas the functional activity of murine MDSCs can be evaluated in antigen-specific assays, the result of human MDSC inhibitory activity is commonly evaluated using crude assays involving pan-T cell stimulation and measuring decreased cell division or cytokine production in the presence of titrated numbers of MDSCs. Again, these biological assays are quite variable and difficult to compare between laboratories (8). One way to try and reduce some variability is for journals to request adherence to standardized methods reporting parameters so that investigators are at least assured that they are using the same approach. Guidelines such as MIATA (minimal information on T cell assays) do exist for this purpose and should be strongly encouraged (30).

### Interventions to Alleviate Suppression

It is emerging that certain chemotherapy agents currently approved for clinical application reduce MDSC levels, and may in fact rely on this facet of their function for a large part of their anti-cancer therapeutic activity. On the other hand, some chemotherapeutic drugs may have the opposite effect and enhance MDSC function. For example, in colon cancer regimes 5-FU has an anti-MDSC effect but Irinotecan a pro-MDSC effect (31). Several chemotherapeutic drugs, even at low doses, as well as those affecting the maturation of myeloid cells (e.g., all-trans retinoic acid, ATRA) may prevent MDSC function (32). A very small ongoing trial combining checkpoint blockade with ATRA treatment in stage IV metastatic melanoma is expected to report next year [2020] (see https://ClinicalTrials.gov/show/NCT02 403778) with an interim analysis available now (33). Anti-inflammatory agents also impinge on the induction and maintenance of MDSC function, and several approaches utilizing drugs modulating inflammatory pathways are ongoing [see (34) for a recent review]. It may prove more effective to target the induction of MDSCs than targeting their suppressive mechanisms and products, as notoriously illustrated by the recent failure of a phase III trial to block IDO (35). While there could have been many reasons for this failure, murine studies demonstrating the strong homeostatic compensation for elimination of tumor-infiltrating MDSCs supports the concept that targeting MDSC induction may be the most effective approach (36). Interest remains high in developing means to successfully modulate this pathway. Given the intrinsic redundancy in immunological feedback control mechanisms, it is likely that multiple pathways will need to be modulated to succeed in this aim, as discussed for example in Ostrand-Rosenberg (14). Of the many additional approaches that could be explored, differentiation of the immature MDSCs away from a suppressive phenotype by physiological induction, for example, of IL-12 by innate immune agonists rather than pharmacological agents, may be a fruitful approach (37).

## Are Levels of MDSCs Informative for Cancer Patient Survival?

The majority of correlative data between MDSCs and clinical outcomes pertains to cancer, where the presence of cells with phenotypes of one type or another have been associated with patient survival in many different tumors including melanoma (38), breast (39), lung (40), and others. These data nearly always refer to assessments on peripheral blood and can only be considered biomarkers for clinical outcome, rather than providing mechanistic inference. However, our studies assessing the ability of patients' PBMCs to respond to candidate tumor-associated antigens *in vitro*, combined with patient MDSC frequency, do show correlations with survival (39, 41). This suggests that suppression of anti-cancer antigen responses due to high levels of MDSCs (but not regulatory T cells) does impinge on clinical outcome. However, changes in levels of MDSCs during checkpoint blockade (with single agent ipilimumab in melanoma) were not associated with overall survival (42). Thus, despite the predictive value of baseline levels of MDSCs, whether or not these changed during treatment with ipilimumab, was not related to responsiveness. We are currently investigating whether the same holds true for the current standard of care that has superceded single agent ipilimumab (anti-PD-1 with or without ipilimumab). There is also evidence that higher levels of MDSCs also result in poorer responses to cancer vaccines (43).

## Are MDSCs Exclusively Pathological?

### MDSCs in Infectious Disease

Combating acute infections requires an inflammatory response which may also cause “collateral” tissue damage, normally repaired once the infection is resolved. However, when the response becomes chronic, homeostasis requires a balance between continued immune surveillance and protection against tissue damage resulting from inflammatory mediators. This may be the reason why inflammatory cytokines and chemokines induce and maintain MDSCs as part of the immune feedback control in chronic infection and why MDSC can induce regulatory T cells (44). This would be a physiological requirement, but could also lead to excessive immunosuppression if unbalanced. What is the evidence for this hypothesis? Prime examples include chronic bacterial (e.g., tuberculosis [TB]) and viral (e.g., Hepatitis C virus [HCV]) infections. While most data are derived from mouse models, myeloid cells with many of the features of MDSCs as defined in cancer patients are found in the blood of TB patients, with reduced numbers after treatment, potentially suggesting a pathological effect of these cells when present in large amounts (45). The same may be true for chronic HCV (46) and HBV infection (47); however, a later study found no relationship between MDSCs and HCV infection (48). This raises the question so prevalent in cancer studies as to the identification and standardization of detection techniques for these cells. Nonetheless, the weight of opinion and data in the literature strongly suggest that MDSCs play important roles in chronic infections, and that these are always or nearly always pathological as more clearly discerned from mouse studies (although the latter are often not at all reliable guides to clinical conditions) (49).

Are there any indications that MDSCs might play beneficial roles in disease? This seems not to be the case for M-MDSCs, but there is some evidence for a beneficial role of PMN-MDSCs in resolving acute HBV infection and preventing liver damage [reviewed in Dorhoi et al. (49)]. Thus, it is conceivable that PMN-MDSCs can have protective effects in limiting tissue damage caused by inflammation in acute infections, but M-MDSCs induced in a chronic inflammatory environment are likely always to exert pathogenic influences. This remains a hypothesis to be rigorously tested.

### MDSCs in Autoimmunity

The role of MDSCs in autoimmunity is hotly debated and controversial (50). Clearly, the normal inflammatory response in infectious disease is self-limiting, but in chronic autoimmune conditions this regulation is disrupted. One factor contributing to this could be a dearth of MDSCs allowing destructive processes to continue. The question is therefore whether MDSCs do have key roles in promoting or maintaining tolerance and whether the mechanism might be via modulating T cell responses. Here again, there are relatively few data. Although only a pilot study, in rheumatoid arthritis, PMN-MDSCs present in the synovial fluid were suggested to contribute to inhibiting autoreactive T cells (51), but clearly did not prevent pathology. The same is true for multiple sclerosis, where there also appears to be no data on M-MDSCs in humans (52). A more recent paper on the rare disease cryopyrin-associated periodic syndromes (CAPS, caused by NLRP3 mutations and consequent overproduction of IL-1β) suggested that MDSCs might act in an anti-inflammatory manner and exert beneficial effects (53). MDSCs have also been implicated in the differential pathology of asthma and COPD (54). Relative to large amounts of work in mice, which may be translatable to humans only with difficulty, there are vanishingly small amounts of data on the role of MDSCs in human autoimmunity, identifying an important unmet need for understanding this pathological process.

### MDSCs in Aging

Considering the strong relationship between MDSCs and cancer, and aging and cancer incidence (55), as well as incidence of other non-communicable age-related diseases, age-related trends pertaining to MDSC expansion and function would be expected. Although, again, little is known about the impact of age on MDSCs in humans, this is indeed the case in mice, which exhibit higher levels of MDSCs in the bone marrow, spleen and peripheral lymph nodes with increasing age (56–58). Importantly for immune function at older age, MDSCs down-regulate L-selectin (CD62L) on naive T cells through their expression of the protease ADAM17 (59). L-selectin on naive T cells is essential for their entry into lymph nodes where they become activated. Aged mice have even lower levels of CD62L on their naive T cells because they have higher levels of MDSCs. Additionally, older mice have a reduced ability to clear MDSCs following experimentally induced expansion (60). Similarly, in humans, compared to young adults (<60 years old), older community-dwelling individuals (61–76 years old) and the frail, institutionalized elderly (67–99 years old) exhibit significantly higher peripheral blood levels of CD33^+^HLA-DR-negative MDSCs, especially the CD11b^+^CD15^+^ subset (5). Higher frequencies of MDSCs in older humans may be limited to the PMN-MDSCs—one small study on 12 people >80 years of age reported higher levels of PMN-MDSCs but not M-MDSCs (6). Interestingly, the mechanisms involved in age-related increases of MDSCs appear to be at least partly determined by well-known aging-associated processes, namely cellular senescence and inflammation, and possibly the skewing of hematopoiesis away from the lymphoid toward the myeloid lineage. Cellular senescence contributes to age-related functional outcomes and systemic inflammation (i.e., the senescence-associated secretory phenotype, SASP) (61). Using a p27 senescence-inducible system, it was found that senescent fibroblasts, via the secretion of proinflammatory cytokines such as IL-6, promoted the local accumulation of MDSCs (62). This coincides with findings from a study of older adults with idiopathic pulmonary fibrosis, suggesting that MDSCs tend to accumulate near fibrotic lesions (63), which are enriched in senescent fibroblasts (64). The role of inflammation has also been demonstrated in naturally aged and in accelerated aging (i.e., ERCC1-deficient) mouse models. Aged and ERCC1-deficient mice exhibited significantly higher frequencies of MDSCs as well as elevated MDSC NF-kB transcriptional activity in the absence of exogenous stimulation (65). Although indirect in humans, there are several lines of evidence connecting MDSCs to the prevalence of age-related diseases, and in some cases, the severity of those diseases. MDSCs have been found to be higher in patients suffering from rheumatoid arthritis and osteoarthritis, especially so for patients with more severe forms of the disease (i.e., elevated CRP and elevated pain levels) (66). This was also observed in patients with amnestic cognitive impairment, where MDSC levels were significantly higher than healthy controls (67). Interestingly, another group found that the frequency of M-MDSCs was significantly higher in the blood of patients with mild forms of Alzheimer's disease (assessed using the clinical dementia rating), but not more severe forms, and that M-MDSCs from these mild cases were more suppressive *ex vivo* (68); the same was true in multiple sclerosis (69). Finally, as mentioned above, MDSCs have been shown to be higher in older adults with idiopathic pulmonary fibrosis (63), as well as incident cases of Parkinson's disease, where they are nearly five-fold above levels in healthy controls (70). Taken together, all these studies strongly suggest a role for aging in the expansion and function of MDSCs, which promote disease in older adults. That being said, further work in humans is vital, particularly the age-related mechanisms that influence MDSCs and the longitudinal relationship between MDSC frequency and age-related disease and other important outcomes.

### MDSCs in Obesity

Obesity and high fat diet (HFD) are established risk factors that contribute to increased cancer incidence, increased tumor progression, and increased cancer mortality (71, 72). Obesity is accompanied by multiple biological changes that contribute to malignancy. One such change is the low-grade inflammation associated with adipose tissue due to the production of TNF, IL-1β, IL-6, and prostaglandin E2 (PGE2). These pro-inflammatory mediators are produced by adipocytes as well as by adipose-infiltrating macrophages (73), and directly impact cancer risk and progression, leading to the concept that obesity-associated inflammation is an important mechanism by which obesity facilitates malignancy (74). The chronic low-grade inflammatory milieu present in obese tissue is similar to the pro-inflammatory environment present in many solid tumors that leads to the induction of MDSCs. Lipids themselves also drive the accumulation and suppressive potency of MDSCs. Mouse and human studies have shown that polyunsaturated fatty acids (PUFAs), such as omega-3 fatty acids, and fatty acid metabolism increase the generation and suppressive activity of MDSCs (75, 76). Given the role of chronic inflammation as a driver of MDSCs and the prevalence of lipid in obese individuals, it is not unexpected that M-MDSC are elevated in obese humans (77).

Studies examining the function of MDSCs in obese individuals have to date only been conducted in mice. Two experimental systems have been used to generate overweight/obese mice: (i) Ob/Ob mice are genetically leptin-deficient and therefore lack appetite control and rapidly become overweight. (ii) Inbred mice fed a HFD consisting of 60% fat become overweight/obese relative to mice kept on a low fat diet (LFD) consisting of 10% fat. In both models M-MDSC and PMN-MDSC levels increase with increasing weight gain (78). As expected, overweight mice on a HFD and with elevated levels of MDSCs have more rapidly growing tumors and more extensive metastatic disease, and their T cells are less activated by antigen. Depletion of MDSCs in HFD mice reverts tumor growth rates to that observed in LFD mice and restores antigen-driven T cell activation, while depletion of both MDSCs and CD4^+^ and CD8^+^ T cells increases tumor growth rate. MDSCs from HFD mice also are more efficient suppressors of antigen-activated T cells, and tumor-infiltrating MDSCs from HFD mice express elevated levels of PD-L1. The latter effect is most likely the result of higher levels of IFNγ in the tumors of HFD mice (79). Elevated levels of MDSC and PD-L1 on MDSC may also provide a broader target for PD-1 checkpoint blockade immunotherapy and explain why PD-1 therapy is more efficacious in obese cancer patients (80).

Increased levels of MDSCs in HFD mice are due to the over-production of leptin, since mice treated with a soluble form of the leptin receptor do not develop high levels of MDSCs (79). However, MDSCs down-regulate leptin since mice depleted of MDSCs contain higher levels of leptin in their blood. Therefore, leptin levels drive the accumulation and function of MDSCs which enhance tumor progression by suppressing antitumor T cell responses. Interestingly, mice on a LFD have decreasing levels of MDSCs as their weight increases, suggesting that LFD is protective against increases in MDSCs, which is typically associated with weight gain (79).

Metabolic dysfunction in the form of elevated fasting glucose and increased insulin resistance is frequent in obese individuals and is characteristic of type 2 diabetes. As expected, Ob/Ob mice and mice on a HFD diet developed elevated fasting glucose levels and increased insulin resistance relative to Ob/+ mice and LFD mice, respectively. Unexpectedly, depletion of MDSCs from HFD mice significantly increased both insulin resistance and fasting glucose levels. Depletion of MDSCs from HFD mice also increased systemic and adipose tissue inflammation (IL-6 and TNF levels, respectively). However, HFD mice depleted of MDSC contained larger parametrial fat pads relative to non-depleted HFD mice. These studies demonstrate that although diet-induced MDSCs can accelerate tumor progression and metastatic disease, at the same time, they also protect against some of the metabolic dysfunction associated with obesity, while increasing adiposity and reducing the inflammation that accompanies adiposity (78). Therefore, in the setting of obesity and nutritional overload, MDSC play a beneficial role in counter-acting conditions that contribute to type 2 diabetes.

### MDSCs in Pregnancy

During pregnancy the mother carries a semi-allogeneic fetus but does not mount an immune response against the embryo's histocompatibility or other antigens. This “maternal-fetal tolerance” has been ascribed to multiple mechanisms including immune suppressive T regulatory cells (Tregs) (81), tolerogenic dendritic cells (82), tryptophan catabolism by IDO (83), and several other factors. Several of these mechanisms are regulated by MDSCs and early studies identified MDSC-like cells in pregnancy. Therefore, it was hypothesized that MDSCs may facilitate maternal-fetal tolerance. Pregnant women have high levels of Arg1 in their blood and in their placenta accompanied by down-regulation of the T cell receptor-associated CD3ζ chain and T cell hypo-responsiveness (84), characteristic effects of MDSCs. Cells with certain characteristics of MDSCs making Arg1, iNOS, and ROS are elevated at all stages of human pregnancy and decrease after parturition (85). Although the data on MDSCs in pregnant women is limited, these findings demonstrate that MDSCs are up-regulated during pregnancy and are consistent with a physiological requirement for MDSC for successful pregnancy (85, 86).

Studies in mice clearly demonstrate that MDSCs are essential for successful pregnancy (76–78). In pregnant mice immature myeloid cells analogous to cancer-induced MDSC accumulate in the placenta and produce the pro-angiogenic molecules matrix metalloproteinase-9 and Bv8 (76). Pregnancy-induced MDSCs tolerize via expression of activated STAT3 (78), and MDSC depletion and reconstitution studies identified implantation as a critical time for MDSC function and maintenance of maternal-fetal tolerance (77).

In addition to their direct effects on T effector cells, MDSC also indirectly impact T cells. Several of these indirect mechanisms have been implicated in inducing maternal-fetal tolerance. For example, maternal-fetal tolerance has been attributed to Tregs but MDSCs are known inducers of Tregs in the setting of cancer (87). This mechanism may well be active in pregnant women (88, 89). Studies of women with spontaneous miscarriages and elective abortions further support a critical role for MDSCs in successful pregnancy. Women experiencing early miscarriages have fewer immune suppressive MDSCs in their blood and endometrium relative to women who have delivered live babies (90). These findings have resulted in clinical trials to induce MDSCs in women with unexplained recurrent miscarriage, apparently with some reported success (91, 92). Collectively the observations in pregnant and aborting women combined with the mechanistic studies in mice demonstrate that MDSCs do play an essential normal physiological role in successful pregnancy by maintaining maternal-fetal tolerance.

### MDSCs in Transplantation

In solid organ transplantation, mostly kidney, reports do suggest changes in levels of MDSCs after allografting (93). The expectation that higher levels of MDSCs might translate to better graft survival does seem to be borne out in several reports. For example, also in human renal transplantation, patients with higher MDSCs experienced less acute graft rejection, and maintained better graft function for a longer period of time (94). Factors influencing the relative levels of MDSCs, proportions of M-MDSCs-vs.-PMN-MDSCs, and the clinical implications of altered levels of these cells under immunosuppression following transplantation are now beginning to be explored (95).

MDSCs may not only directly inhibit effector T cells responsible for graft rejection, but also amplify Tregs (96). Interest in manipulating MDSCs to further transplantation tolerance in humans, as opposed to mouse models, is only recently becoming widespread, and most experience has been gained in cancer where efforts have been directed toward inhibiting MDSCs, not stimulating them (97). In mice, enhancing MDSC induction may confer benefit. For example in a skin transplant model, a combination of G-CSF and IL-2 coupled to an anti-IL 2 antibody increased MDSC (as well as Treg) levels and extended graft survival (98). In the naturally more tolerogenic human liver transplant setting, one mechanism by which tolerance is induced seems to be by stimulation of MDSCs (99). In a different clinical transplantation setting, the role of MDSCs has proven more equivocal. Although MDSCs may be beneficial in reducing graft-vs.-host disease (GVHD) in hematopoietic stem cell (HSC) transplantation, at the same time they can be inhibitory for T cell reconstitution and thus mediate negative effects [reviewed in (100)]. However, even in solid organ transplantation, MDSCs are a double-edged sword and can contribute to excessive immunosuppression (101). Nonetheless, efforts to control their induction not only by using agents known to enhance MDSCs in the cancer field (i.e., pro-inflammatory factors such as TNF (102), or G-CSF (103), or immune modulators such as dexamethasone (104), but also by novel approaches such as the use of cannabinoids (105), are ongoing. In murine HSC transplantation, a report of successfully applying *in vitro*-generated MDSCs to prevent GVHD at the same time allowing retention of CD8+ cytotoxic T cell effector function to maintain anti-cancer activity (106) raises hope that this outcome may also be achieved in humans and in solid organ transplantation.

## Conclusions

**Figure 1:**
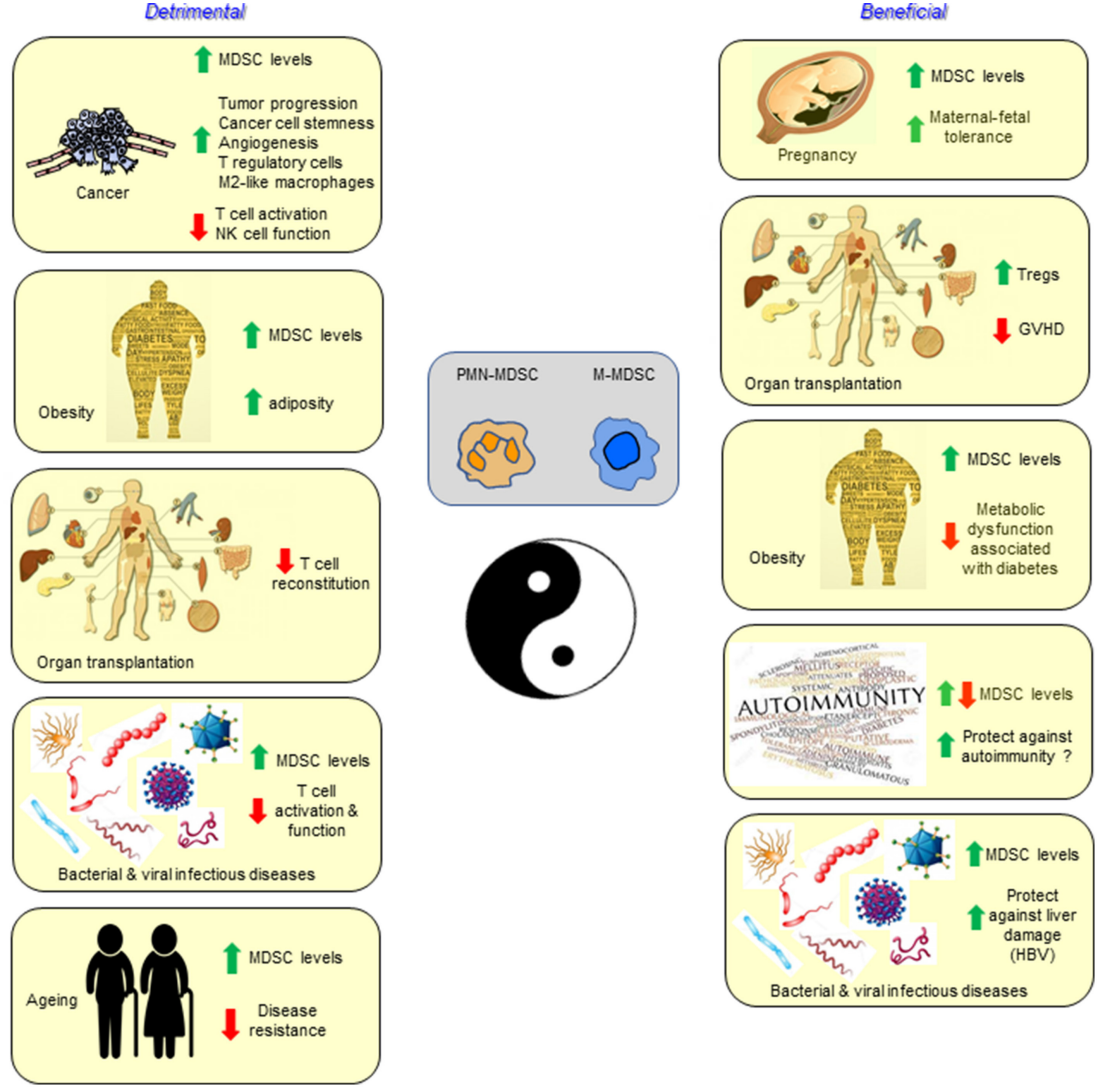
Myeloid-derived suppressor cells are best characterized and studied in the setting of cancer, but also accumulate and function in infectious diseases, autoimmunity, aging, pregnancy, transplantation, and obesity. In most conditions the MDSCs have a detrimental effect, while in other settings they may contribute to the health of the individual.

The class of immune cells designated MDSCs is unequivocally important in dampening immunity in a wide range of cancers, and also in other pathological conditions involving chronic inflammation (Figure 1). However, there is also some evidence of a potentially beneficial effect in the iatrogenic situation of solid organ transplantation, as well as the parallel physiological “transplant” situation of pregnancy, and in combating some of the metabolic dysfunction associated with the pathology of obesity. We thus conclude that unlike Tregs, MDSCs are not likely to play a major role in the normal feedback control of immune responses with the single possible exception of their involvement in fetal tolerance.

## Author Contributions

GP, CV, and SO-R all drafted, reviewed, edited, and approved the manuscript.

### Conflict of Interest Statement

The authors declare that the research was conducted in the absence of any commercial or financial relationships that could be construed as a potential conflict of interest.
